# Limited Access to a High Fat Diet Alters Endocannabinoid Tone in Female Rats

**DOI:** 10.3389/fnins.2018.00040

**Published:** 2018-02-02

**Authors:** Valentina Satta, Maria Scherma, Fabiana Piscitelli, Paolo Usai, M. Paola Castelli, Tiziana Bisogno, Walter Fratta, Paola Fadda

**Affiliations:** ^1^Division of Neuroscience and Clinical Pharmacology, Department of Biomedical Sciences, University of Cagliari, Cagliari, Italy; ^2^Endocannabinoid Research Group, Institute of Biomolecular Chemistry, National Research Council, Pozzuoli, Italy; ^3^Department of Medical Sciences and Public Health, University of Cagliari, Cagliari, Italy; ^4^Department of Medicine, Campus Bio-Medico University of Rome, Rome, Italy

**Keywords:** binge eating disorder, high fat diet, anandamide, 2-arachidonoyl glycerol, cannabinoid type-1 receptors

## Abstract

Emerging evidence suggest an impaired endocannabinoid activity in the pathophysiology of binge eating disorder (BED). Herein, we investigated whether endocannabinoid tone could be modified as a consequence of dietary-induced binge eating in female rats. For this purpose, brain levels of the endocannabinoids anandamide (AEA) and 2-arachidonoyl glycerol (2-AG), as well as two endocannabinoid-like lipids, oleoylethanolamide (OEA) and palmitoylethanolamide (PEA), were assessed in different brain areas involved in the hedonic feeding (i.e., prefrontal cortex, nucleus accumbens, amygdala, hippocampus, and hypothalamus). The brain density of cannabinoid type-1 receptors (CB_1_) was also evaluated. Furthermore, we determined plasma levels of leptin, ghrelin, and corticosterone hormones, which are well-known to control the levels of endocannabioids and/or CB1 receptors in the brain. To induce binge eating behavior, rats were subject to an intermittent and limited access to a high fat diet (HFD) (margarine). Three experimental groups were used, all with *ad libitum* access to chow: control (CTRL), with no access to margarine; low restriction (LR), with 2 h margarine access 7 days/week; high restriction (HR), with 2 h margarine access 3 days/week. Bingeing was established when margarine intake in the HR group exceeded that of the LR group. Our results show that, compared to CTRL, AEA significantly decreased in the caudate putamen, amygdala, and hippocampus of HR group. In contrast, 2-AG significantly increased in the hippocampus while OEA decreased in the hypothalamus. Similar to the HR group, AEA and OEA decreased respectively in the amygdala and hypothalamus and 2-AG increased in the hippocampus of LR group. Moreover, LR group also had AEA decreased in the prefrontal cortex and increased in the nucleus accumbens. In both groups we found the same reduction of CB_1_ receptor density in the prefrontal cortex compared to CTRL. Also, LR and HR groups showed alterations in both ghrelin and corticosterone levels, while leptin remained unaltered. In conclusion, our findings show a modified endocannabinoid tone due to margarine exposure, in several brain areas that are known to influence the hedonic aspect of food. Even if not uniquely specific to binge eating, margarine-induced changes in endocannabinoid tone could contributes to the development and maintenance of this behavior.

## Introduction

Binge eating disorder (BED) is one of the most common eating disorders and is characterized by recurrent and persistent episodes of compulsive overeating of certain foods (binge eating), typically highly palatable foods rich in calories, that are not inevitably motivated by hunger or metabolic needs (APA, 2013). BED is more prevalent in young people, with a lifetime prevalence of 1.4% (females:males, 6:4) and in the majority of patients is accompanied by significant psychiatric comorbidities (Hudson et al., 2007; Kessler et al., 2013; Guerdjikova et al., 2017). Additionally, this aberrant eating behavior is not always followed by regular use of compensatory behaviors, such as laxative consumption, vomiting, or excessive exercise; consequently, patients often develop obesity (Guerdjikova et al., 2017). The pathophysiology underlying BED is not yet completely clarified and new specific knowledge are needed to better understand the neuronal processes which contribute to the course of this disease. Several line of evidence suggest that a dysfunction in the mesocorticolimbic system, that represent the principal neural pathway that drive hedonic feeding, could be a main mechanism associated with development of this disorder (Davis and Carter, 2009; Dichter et al., 2012; Witt and Lowe, 2014). This pathway consist of subpopulations of dopaminergic neurons, originating in the ventral tegmental area and pars compacta of the substantia nigra, which project to the nucleus accumbens, as well as to other limbic structures, such as the amygdala, hippocampus, and prefrontal cortex (Kenny, 2011; Hutson et al., 2018). Moreover, all these areas are highly connected to the hypothalamus, and many molecules involved in homeostatic hypothalamic regulation of feeding, such as leptin and ghrelin, also play an important role in the hedonic and motivational components of food (Monteleone and Maj, 2013; Murray et al., 2014).

The endocannabinoid system is composed of cannabinoid type-1 (CB1) and -2 (CB2) receptors, their endogenous ligands, called endocannabinoids [anandamide (AEA) and 2-arachidonoylglycerol (2-AG)], enzymes that produce and metabolize endocannabinoids, and transporters (Di Marzo, 2009). Altogether, components of the endocannabinoid system participate in the control of a wide range of physiological processes, among which the hedonic aspects of eating (Lau et al., 2017). Consistent with this, various human and animal studies have shown that endocannabinoids stimulate food intake particularly increasing the consumption of palatable foods by acting on CB_1_ receptors expressed within the mesocorticolimbic regions (D'Addario et al., 2014; Jager and Witkamp, 2014; Lau et al., 2017). Moreover, endocannabinoids interact with other signaling pathways involved in the hedonic aspect of food, including the dopaminergic system (D'Addario et al., 2014; Lau et al., 2017). In addition to classical endocannabinoids, the endocannabinoid-like lipids such as palmitoylethanolamide (PEA) and oleoylethanolamide (OEA), which act as ligands for the peroxisome proliferator-activated receptor-α (PPARα), also play an important role in modulating eating behavior by acting as a mediator of satiety (Fu et al., 2003; Lo Verme et al., 2005; Romano et al., 2015). Moreover, OEA seems also to interact with hedonic signaling reducing the consumption of high-calorie foods (Brown et al., 2017).

Defects in endocannabinoid signaling have been implicated in development of BED (Scherma et al., 2015). For example, elevated plasma levels of AEA were found in overweight/obese patients with BED (Monteleone et al., 2005). Moreover, overweight and obese subjects reportedly have an increased frequency of a naturally occurring missense polymorphism in the gene that encodes the AEA hydrolyzing enzyme fatty acid amide hydrolase, which may attenuate endocannabinoid inactivation and increase endocannabinoid signaling (Sipe et al., 2005). Since the endocannabinoid AEA is an essential component of brain mechanisms controlling reward, elevation of the endocannabinoid tone could reinforces the hedonic properties of palatable food, thus favoring food addiction and perpetuating binge eating behavior (Monteleone et al., 2005).

Different animal models of binge eating have been developed (Corwin and Buda-Levin, 2004; Corwin et al., 2011). In the limited access model, binge eating behavior is induced in rats by providing sporadic and time-limited access (e.g., 2 h, 3 days/week) to an optional source of high fat diet (HFD) with continuously available chow (Corwin et al., 1998; Dimitriou et al., 2000). These feeding schedules elicit compulsive overeating of the HFD that remains stable over prolonged periods of time. Moreover, since animals were never food deprived, this condition was similar to binging humans who eat in the absence of hunger (Marcus and Kalarchian, 2003). Using this protocol, we recently demonstrated that the CB1 receptor inverse agonist/antagonist rimonabant decreases binge eating behavior in female rats by selectively reducing consumption of the HFD (margarine) upon which they binge (Scherma et al., 2013). Although the mechanism through which rimonabant exert its effects on binge eating behavior is still unclear, this finding suggest that it may suppress the hedonic response to food rather than just hunger, supporting the hypothesis of an altered endocannabinoid tone in the neural pathways that drive hedonic feeding in binging rats. On the other hand, it is well established that tissue concentrations of endocannabinoids and related lipid-derived molecules as well as CB receptor expression are influenced by several factors, including dietary conditions such as prolonged exposure to HFD (Jager and Witkamp, 2014; Brown et al., 2017; Zamberletti et al., 2017). In keeping with this, we hypothesized that exposure to HFD, which promote binge eating, would alter the endocannabinoid tone that could contribute to sustainment of its compulsive intake. Thus, the present study investigated the impact of HFD-induced binge eating behavior on endocannabinoid tone, specifically in mesocorticolimbic brain areas such as the prefrontal cortex, striatum, amygdala and hippocampus, as well as in the hypothalamus. For this purpose, brain levels of two endocannabinoids AEA and 2-AG and two endocannabinoid-like lipids OEA and PEA, as well as CB1 receptor brain density, were assessed.

Human studies have shown that many peripheral hormones, like leptin and ghrelin, are disrupted in patients with BED (Culbert et al., 2016). Moreover, most of these patients have dysfunctional hypothalamic-pituitary-adrenal axis activity and different studies have hypothesized that elevated glucocorticoid levels may sustain compulsive overeating (Lavagnino et al., 2014; Razzoli et al., 2017). Finally, the levels of endocannabioids and/or CB_1_ receptors in the brain are under the control of these hormones (Di Marzo et al., 2001; Balsevich et al., 2017). For this reason, their plasma levels were also measured.

## Materials and methods

### Animals

Thirty six female Sprague Dawley rats (Envigo, Italy) weighing 185–200 g at the start of the study (60–65 days-old) were used. Female rats were chosen because BED is more common in female than in man (Preti et al., 2009). Following arrival, animals were individually housed in a climate-controlled animal room (21 ± 2°C and 60% humidity) under a reversed 12 h light/12 h dark cycle (lights on at 24:00). All rats had *ad libitum* access to standard rat chow and water. All procedures and experiments were carried out in an animal facility according to Italian (D.L. 26/2014) and European Council directives (63/2010) and in compliance with approved animal policies by the Ethical Committee for Animal Experiments at the University of Cagliari (Sardinia, Italy) and the Italian Department of Health (286/2016). All possible efforts were made to minimize animal pain and discomfort, as well as reduce the number of experimental subjects.

### Diets

Standard rat chow (Safe, France) consisted of 3% kcal from fat, 61% kcal from carbohydrate, 16% kcal from protein, 20% moisture, and containing 2.9 kcal/g. HFD (margarine; Gradina Unilever Italia Mkt., Italy) consisted of 70% kcal from fat, <1% kcal from carbohydrate, and containing 6.5 kcal/g).

### Induction of binge eating behavior

**Figure 1:**
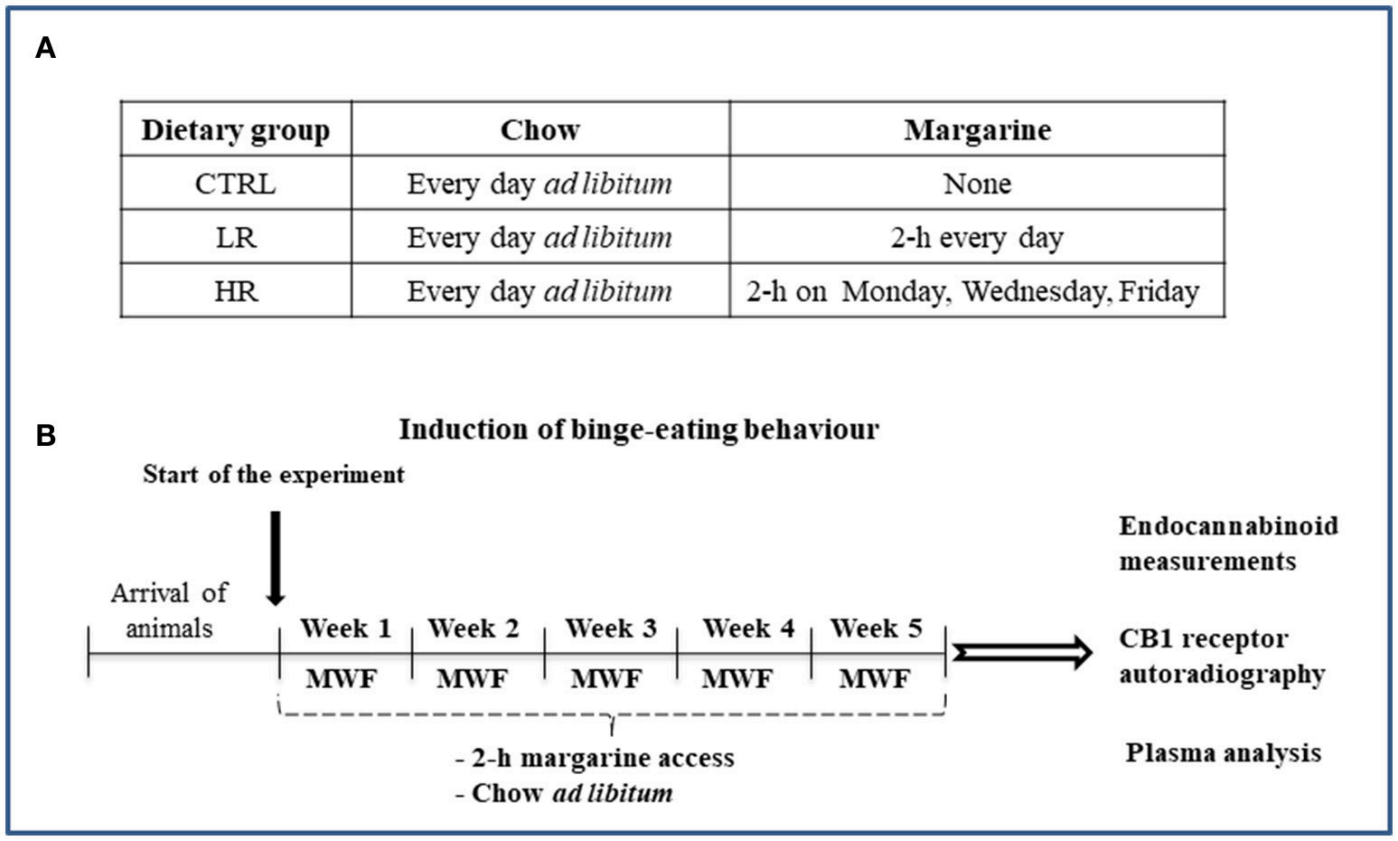
Schematic representation of the experimental timeline: **(A)** Dietary groups; **(B)** Induction of binge eating behavior.

Binge eating behavior was induced by using the limited access protocol as previously described (Scherma et al., 2013; Satta et al., 2016). Briefly, after 1 week of acclimatization, margarine was provided during a single overnight period to prevent neophobia. Rats were then matched by body weight and divided into three dietary groups (*n* = 12 per diet group; Figure 1A):

- Control (CTRL): standard chow and water were available *ad libitum*. Margarine was not provided at any time during the study.
- Low restriction (LR): standard chow and water were available *ad libitum*. In addition, animals were given 2 h access to a separate bowl of margarine which was introduced into the home cage every day of the week.
- High restriction (HR): standard chow and water were available *ad libitum*. In addition, animals were given 2 h access to a separate bowl of margarine which was introduced into the home cage on Mondays, Wednesdays, and Fridays.

In this model, as access to the fat decreases, consumption of the fat increases when it is provided, thus binging is operationally defined when margarine-intake in the HR group exceeds that of the LR group (Corwin et al., 2011).

Animals allowed to eat margarine were given access to it in the light phase starting 2 h prior to the start of the dark cycle. All rats were maintained on their respective diets throughout the entire study (5 weeks). Margarine was measured for both LR and HR groups on Mondays (M), Wednesdays (W), and Fridays (F) by weighing it before and after the 2 h access period. Standard chow was measured for all dietary groups on M, W, and F by weighing it before and after the 2 h access period as well as 22 h after 2 h access [on Tuesday (T), Thursday (Th) and Saturday (S)], in order to determinate the 24 h cumulative food intake. Body weight from all dietary groups was recorded weekly. At the end of the 2 h margarine access period on the last day of the study period (week 5), rats were sacrificed and samples were collected to carry out our analysis of the levels of endocannabinoids and CB1 receptor density as well as of plasma levels of leptin, ghrelin and corticosterone (Figure 1B).

### Lipid extraction and endocannabinoid measurements

Upon sacrifice, rat brains from each dietary group (CTRL, LR and HR, *n* = 6 per group) were quickly removed and the cerebral areas of interest (prefrontal cortex; striatum: caudate putamen and nucleus accumbens; hippocampus; amygdala; and hypothalamus) were obtained by regional dissection on ice, immediately frozen in liquid nitrogen, and stored at −80°C until processing. As previously described (Marsicano et al., 2002), tissues were dounce homogenized and extracted with 2:1:1 (v/v) chloroform:methanol:Tris-HCl (50 mM, pH 7.4) containing internal deuterated standards for AEA (d8-AEA), 2-AG (d5-2-AG), OEA (d4-OEA), and PEA (d4-PEA), and then quantified by isotope dilution (Cayman Chemicals, MI, USA). The lipid-containing organic phases were then purified by open-bed chromatography on silica, and fractions were obtained by eluting the column with 99:1, 90:10, and 50:50 (v/v) chloroform:methanol. Fractions eluted with 90:10 (v/v) chloroform: methanol were collected, excess solvent evaporated with a rotating evaporator, and aliquots analyzed by isotope dilution liquid chromatography (LC)/atmospheric pressure chemical ionization/mass spectrometry (MS) carried out in selected ion monitoring mode using a Shimadzu high performance LC apparatus (LC-10ADVP) coupled to a Shimadzu (LC-MS-2020) quadrupole MS via a Shimadzu Atmospheric Pressure Chemical Ionization interface. MS detection was performed using m/z values of 356 and 348 [molecular ions (M)+1 values for d8-AEA and AEA], 384 and 379 (M+1 values for d5-2-AG and 2-AG), 330 and 326 (M+1 values for d4-OEA and OEA), and 304 and 300 (M+1 values for d4-PEA and PEA). AEA, 2-AG, OEA, and PEA levels were calculated based on the ratio of their peak area to that of the internal deuterated standard. Lipid amounts expressed as pmol were then normalized per g or mg of wet tissue.

### CB_1_ receptor autoradiography

**Figure 2:**
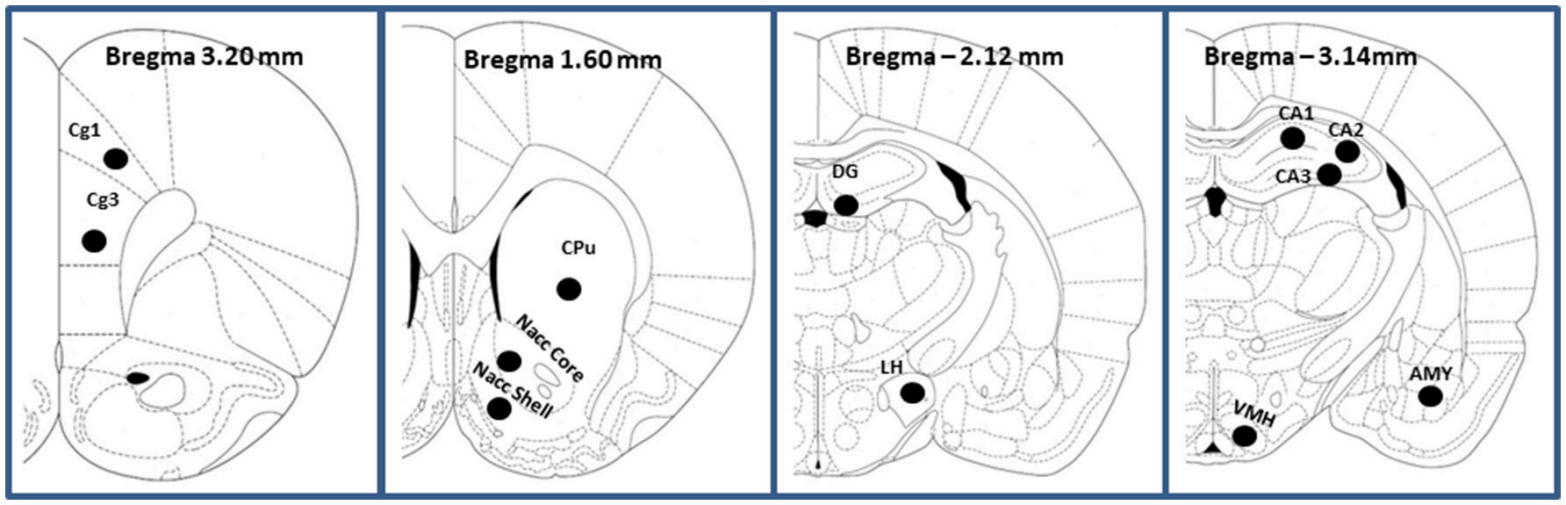
Schematic representation (adapted from Paxinos and Watson, 1998; the Rat Brain in Stereotaxic Coordinates) of the prefrontal cortex (Cingulate cortex Cg1 and Cg3), caudate-putamen (CPu), Nucleus accumbens (Nacc Core and Nacc Shell), Hippocampus (CA1 field of Ammon's horn; CA2, field of Ammon's horn; CA3, field of Ammon's horn; DG, dentate gyrus of hippocampus), Amygdala (AMY) and Hypotalamus (lateral LH and ventro-medial VHM).

Rats from each dietary group (CTRL, LR and HR, *n* = 6 per group) were sacrificed and brains were rapidly removed and frozen in methylbutane (Sigma Aldrich) kept at −35°C on dry ice and then stored at −80°C before being sliced in a cryostat. Coronal sections (12–16 μm thick) were prepared with a cryostat at −20°C, thaw-mounted onto Superfrost Plus slides (Clini-Lab s.r.l., Conselve, Italy), and stored at −20°C until use. Brain regions selected for analysis, according to the Atlas of Paxinos and Watson (1998), included the prefrontal cortex: cingulate cortex areas 1 and 3 (Cg1 and Cg3) (AP: +3.2), the caudate-putamen (CPu), nucleus accumbens core and shell (Nacc core and Nacc shell; AP: +1.60), cornu ammonis 1–3 (CA1, CA2, and CA3) fields of hippocampus, dentate gyrus (DG) of hippocampus, AP: from −2.12 to −3.14), amygdala (AMY; AP: −2.12 to −3.14), hypothalamus [ventromedial (VHM) and lateral (LH); AP: −2.14) (Figure 2). [3H]-CP 55,940 binding autoradiography was performed as previously described by Castelli et al. (2014). Briefly, tissue slides were incubated at 37°C for 2.5 h in 50 mM Tris-HCl (pH 7.4) containing 5% bovine serum albumin and 10 nM [3H]CP55940 (specific activity, 131.8 Ci/mmol; Perkin Elmer Life Sciences, Milan, Italy). Non-specific binding was determined in adjacent brain sections in the presence of 10 μM unlabeled CP55940. Following incubation, tissue slides were rinsed twice at 4°C for 2 h in ice-cold Tris-HCl buffer (50 mM, pH 7.4) with 1% bovine serum albumin, once for 5 min with 50 mM Tris-HCl, dipped in ice-cold deionized water, and then air-dried. Dried tissue sections and slide-mounted [3H]micro-scales standards (RPA 501 and 505; Amersham, USA) for [3H](-)-CP55940 autoradiography were placed in a Fujifilm BAS cassette with a BAS-5000 imaging plate. The resulting images were analyzed with a Fujifilm-BAS 5000 imaging system (Automatic Image Data Analyzer, Ray test, Wilmington, NC, USA), and optical densities were transformed into levels of bound radioactivity (fmol/mg protein) with gray values generated by co-exposed [3H].

### Plasma analysis

Trunk blood from each dietary group (CTRL, LR, and HR, *n* = 6 per group) was collected into K3EDTA tubes, centrifuged at 3,000 × g for 15 min at 4 ± 2°C, and then plasma was stored at−20 °C till analysis. To avoid breakdown of active ghrelin which is extremely unstable in plasma, trunk blood was collected into K3EDTA tubes previously prepared by adding enough of the protease inhibitor 4-benzenesulfonyl fluoride HCl (TE Pro Service, Italy). Plasma levels were measured using a commercially available enzyme-linked immunosorbent assay (ELISA) kit according to the manufacturer's protocols [EZRL-83K/Rat Leptin ELISA, EMD Millipore, St. Charles, MI, USA; EZRGRA-90K Rat/Mouse Ghrelin (active) ELISA, EMD Millipore, St. Charles, MI, USA; Corticosterone Elisa Kit ADI-900-097, Enzo Life Sciences, Lausen, Switzerland].

### Statistical analysis

Data related to margarine and chow intake were expressed as mean kcal of ± SEM and were analyzed by two-way analysis of variance (ANOVA) for repeated measures with dietary group and time (days of study or week) as factors and time as a repeated factor. Data from body weight are expressed as mean in g ± SEM and were analyzed by two-way ANOVA with dietary group and week as main factors and week as a repeated factor. Autoradiography data are expressed as means ± SEM and were analyzed by one-way ANOVA with dietary group as a between-subjects factor. Endocannabinoid levels were expressed as means ± SEM and were analyzed by one-way ANOVA with dietary group as a between-subjects factor. *Post-hoc* Newman–Keuls multiple comparison or Bonferroni test were completed when appropriate. In all cases, differences with a *P* < 0.05 were considered significant.

## Results

### Induction of binge eating behavior

**Figure 3:**
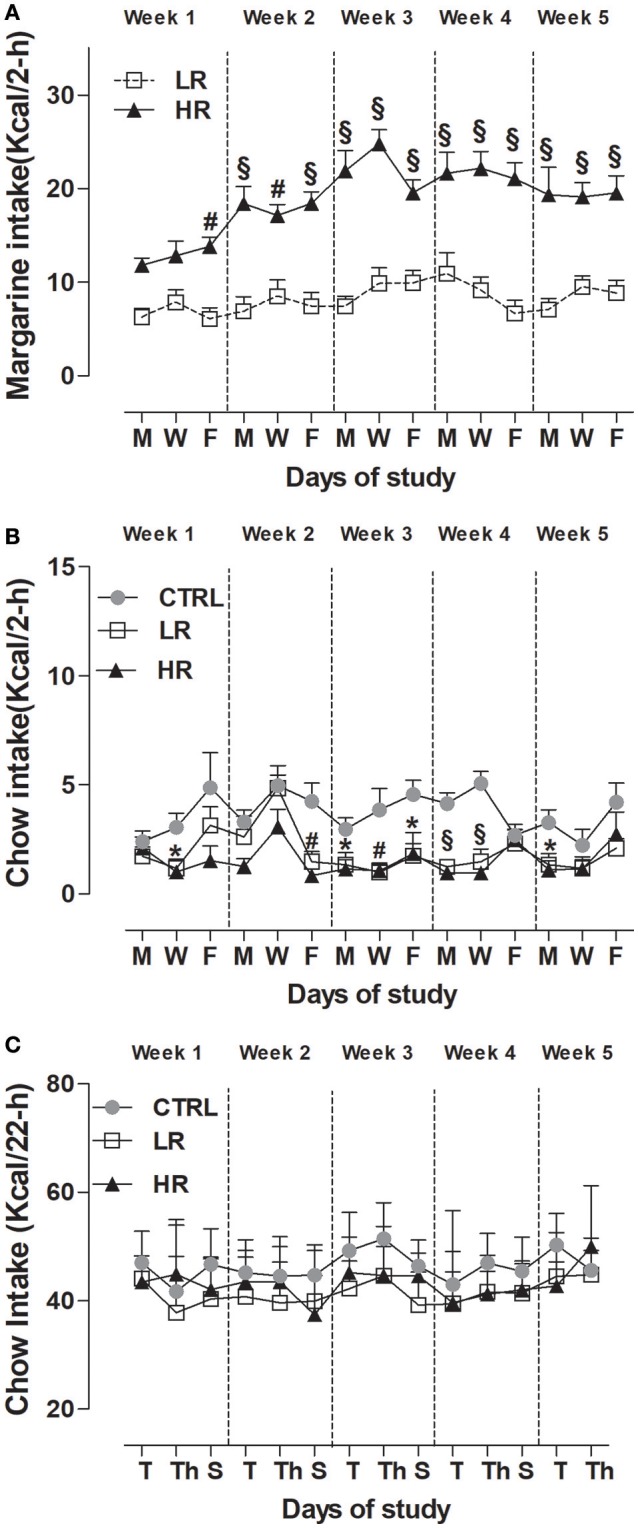
Induction of binge-type eating. Data are presented as mean kcal ± SEM (*n* = 12 rats per group). **(A)** Margarine intake during the limited (2 h) access: HR group consumed more margarine compared to LR group (Two-way ANOVA: ^#^*P* < 0.01 and ^§^*P* < 0.001, Bonferroni test vs. LR group); **(B)** Chow intake during the limited (2 h) access: LR group and HR group consumed less chow compared to CTRL group (one way ANOVA: ^*^P < 0.05, ^#^*P* < 0.01 and ^§^*P* < 0.001, Newman–Keuls test vs. CTRL group). **(C)** Chow intake after the limited (2 h) access: no difference was found between groups.

As expected from previous studies (Scherma et al., 2013; Satta et al., 2016), sporadic and limited access to margarine leads HR group to consume a significantly higher amount of margarine than LR group (Figure 3A). Two-way ANOVA showed a significant dietary group x time interaction [*F*_(14, 308)_ = 3.09, *P* < 0.001] and *post-hoc* analysis revealed a significant difference by the first week of the study (*P* < 0.01 and *P* < 0.001).

With respect to chow consumption during the 2 h limited access period, two-way ANOVA revealed a significant effect of dietary group [*F*_(2, 462)_ = 32.51, *P* < 0.0001] and subsequent individual one-way ANOVA within each day, showed that both LR and HR groups consumed significantly less chow than CTRL [week 1: [W] *F*_(2, 33)_ = 5.560, *P* < 0.01; week 2: [F] *F*_(2, 33)_ = 9.997, *P* < 0.001; week 3: [M] *F*_(2, 33)_ = 3.919, *P* = 0.0297, [W] *F*_(2, 33)_ = 6.527, *P* < 0.05, [F] *F*_(2, 33)_ = 4.485, *P* < 0.05; week 4: [M] *F*_(2, 33)_ = 21.26, *P* < 0.0001, [W] *F*_(2, 33)_ = 20.33, *P* < 0.0001; week 5: [M] *F*_(2, 33)_ = 5.498, *P* < 0.01] (Figure 3B). On the contrary, no significant difference was found between groups on chow-intake measured 22 h that followed the 2 h limited access period: Two-way, dietary group × time interaction *F*_(26, 429)_ = 1,493, *P* = *ns*; dietary group *F*_(2, 33)_ = 3.27, *P* = *ns* (Figure 3C).

When looking at the total caloric intake (chow + margarine) consumed by each dietary group during the 24 h period calculated as 1-block week (M/T, W/Th, F/S), two-way ANOVA detected a significant dietary group x time interaction [*F*_(8, 132)_ = 2.14, *P* < 0.05], and *post-hoc* analysis showed the HR group had a higher total intake than the LR and CTRL groups from the first week of the study (Table 1).

**Table 1:** Cumulative food intake: data are presented as mean kcal/24 h (1-block week: M/T, W/Th, F/S) ±SEM (*n* = 12 per group).

**Dietary-group**	**Week 1**	**Week 2**	**Week 3**	**Week 4**	**Week 5**
CTRL	49.26 ± 1.99	48.86 ± 1.62	53.02 ± 1.53	49.85 ± 2.65	48.52 ± 1.87
LR	50.49 ± 2.24	50.70 ± 2.62	50.55 ± 1.62	49.89 ± 1.85	53.24 ± 1.83
HR	58.47 ± 2.64^*^	64.37 ± 2.57^§^	67.82 ± 2.57^§^	65.10 ± 2.57^§^	66.79 ± 2.72^§^

HR group displayed higher total intake compared to CTRL and LR groups (two-way ANOVA:

* P < 0.05 and

§ *P < 0.001, Bonferroni test vs. CTRL and LR groups)*.

Considering body weight, Two-way ANOVA revealed a significant dietary group x time interaction [*F*_(8, 132)_ = 2.04, *P* < 0.05]. *Post-hoc* analysis indicated the HR group weighed more than the CTRL group by week 4; no difference between CTRL and LR rats was found (Table 2).

**Table 2:** Body weight: data are presented as mean g ± SEM (*n* = 12 per group).

**Dietary-group**	**Week 1**	**Week 2**	**Week 3**	**Week 4**	**Week 5**
CTRL	226.1 ± 3.73	238.1 ± 3.87	245.0 ± 3.91	250.8 ± 3.85	256.8 ± 4.02
LR	229.3 ± 4.11	242.4 ± 5.08	249.9 ± 4.74	257.6 ± 5.49	267.4 ± 6.54
HR	231.1 ± 4.49	244.6 ± 5.53	255.4 ± 5.62	262.1 ± 5.10^*^	269.2 ± 5.14^*^

HR group weighed more compared to CTRL (two-way ANOVA:

* *P < 0.05, Bonferroni test vs. CTRL group)*.

Finally, when we normalized total caloric intake to body weight over a 24 h period calculated as 1-block week (M/T, W/Th, F/S) (kcal/kg), two-way ANOVA revealed a significant effect of dietary group [*F*_(2, 132)_ = 12.66, *P* < 0.0001]. Subsequent individual one-way ANOVA within each week, showed that the HR group exhibited greater normalized total caloric intake across week compared both LR and CTRL groups [week 1: *F*_(2, 33)_ = 4.168, *P* < 0.05; week 2: *F*_(2, 33)_ = 9.224, *P* < 0.001; week 3: *F*_(2, 33)_ = 14.44, *P* < 0.0001; week 4: *F*_(2, 33)_ = 8.875, P < 0.001; week 5: *F*_(2, 33)_ = 11.01, *P* < 0.001; Table 3].

**Table 3:** Normalized total caloric intake to body weight over a 24 h period calculated as 1-block week (M/T, W/Th, F/S) (kcal/kg): data are presented as mean ± SEM (*n* = 12 per group).

**Dietary-group**	**Week 1**	**Week 2**	**Week 3**	**Week 4**	**Week 5**
CTRL	216.51 ± 10.22	206.2 ± 8.02	217.6 ± 7.90	199.2 ± 10.60	189.6 ± 8.12
LR	220.4 ± 10.11	210.4 ± 21.35	203.8 ± 8.60	194.7 ± 8.68	200.5 ± 8.11
HR	254.3 ± 10.21^*^	264.6 ± 11.34^§^	267.3 ± 9.78^#^	250.1 ± 11.49^#^	250.2 ± 12.33^§^

HR group displayed higher normalized total caloric intake compared to CTRL and LR groups (one-way ANOVA:

* P < 0.05 Newman–Keuls multiple comparison test vs. CTRL;

§ P < 0.001 and

# *P < 0.0001 Newman–Keuls multiple comparison test vs. CTRL and LR groups)*.

### Effect of binge eating behavior on endocannabinoid levels

**Figure 4:**
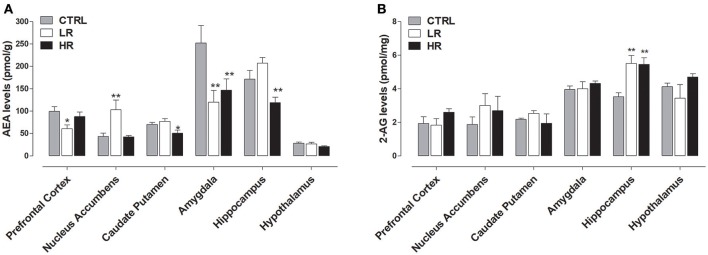
Endocannabinoid levels: data are presented as mean ± SEM (*n* = 6 rats per group). **(A)** AEA: HR group showed a decrease in Caudate Putamen, Amygdala and Hippocampus compared to CTRL group (one-way ANOVA: ^*^P < 0.05 and ^**^P < 0.01, Newman–Keuls test vs. CTRL); LR group showed a decrease in Prefrontal Cortex and Amygdala as well as an increase in the Nucleus accumbens compared to CTRL group (one-way ANOVA: ^*^P < 0.05 and ^**^P < 0.01, Newman–Keuls multiple comparison test vs. CTRL group). **(B)** 2-AG: both LR and HR groups showed an increase compared to CTRL group in the hippocampus (one-way ANOVA: ^**^P < 0.01, Newman–Keuls test vs. CTRL group).

As shown in Figure 4A, endocannabinoids levels were affected by exposure to margarine and one-way ANOVA revealed a significant effect of dietary group. Specifically, decreased AEA levels were found in the caudate putamen [*F*_(2, 15)_ = 8.727, *P* < 0.01], hippocampus [*F*_(2, 15)_ = 8.727, *P* < 0.01], and amygdala [*F*_(2, 15)_ = 5.161, *P* < 0.05] of the HR group compared to CTRL group that never had access to margarine. Similar to HR group, decreased AEA levels were also found in the amygdala [one-way ANOVA *F*_(2, 15)_ = 8.727, *P* < 0.01] of the LR group. Moreover, LR group also had AEA decreased in the prefrontal cortex [one-way ANOVA *F*_(2, 15)_ = 4.195, *P* < 0.05] and increased in the nucleus accumbens when compared to both CTRL and HR groups [one-way ANOVA: *F*_(2, 15)_ = 6.75, *P* < 0.01]. Margarine exposure also affected 2-AG levels since an increase was found in the hippocampus of both LR and HR groups compared to the CTRL group [one-way ANOVA: *F*_(2, 15)_ = 8.467, *P* < 0.01; Figure 4B].

### Effect of binge eating behavior on endocannabinoid-like lipid levels

**Figure 5:**
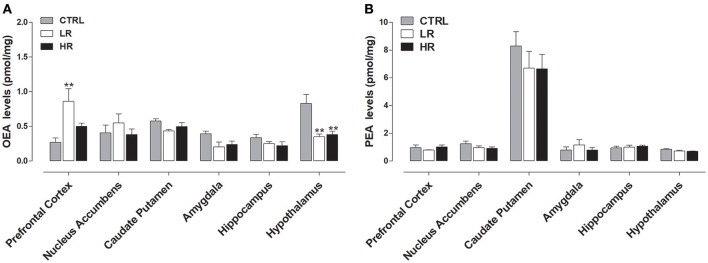
Endocannabinoid-like compounds levels: data are presented as mean ± SEM (*n* = 6 rats per group). **(A)** OEA: both LR and HR groups showed a decrease in the hypothalamus compared to CTRL group (one-way ANOVA: ^**^P < 0.01, Newman–Keuls test vs. CTRL group). LR group showed an increase in the Prefrontal Cortex compared to CTRL group (one-way ANOVA: ^**^P < 0.01, Newman–Keuls test vs. CTRL group). **(B)** No changes in the levels of PEA were found between all three experimental groups.

Figure 5A shows that margarine exposure increased OEA levels within the prefrontal cortex of the LR group compared to CTRL rats [one-way ANOVA: *F*_(2, 15)_ = 6.431, *P* < 0.01] and decreased OEA in the hypothalamus of both LR and HR groups relative to CTRL rats [one-way ANOVA: *F*_(2, 15)_ = 9.976, *P* < 0.01]. No significant changes in OEA levels were observed in the other brains areas analyzed. Finally, no changes in the levels of PEA were found between all three dietary groups (Figure 5B).

### Effect of binge eating behavior on CB1 receptor density

**Figure 6:**
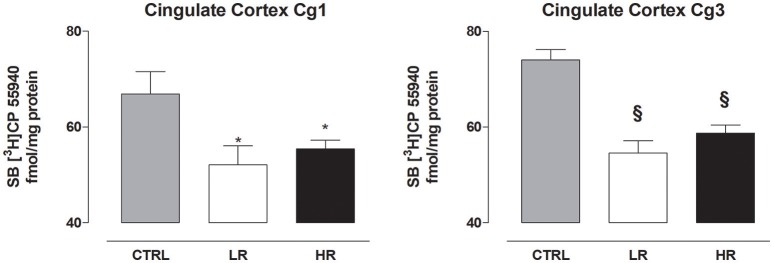
CB_1_ receptor density in the cingulate cortex Cg1 and Cg3. Data are expressed as mean fmol/mg protein of [3H]CP55940 ± SEM (*n* = 6 per group). Both LR and HR groups showed a decrease in the Cg1 and Cg3 compared to CTRL group (one-way ANOVA: ^*^P < 0.05 and ^§^*P* < 0.001, Newman–Keuls test vs. CTRL group).

In the prefrontal cortex, individual one-way ANOVA revealed significantly lower levels of CB1 in Cg1 and Cg3 of LR and HR group compared to CTRL [Cg1: *F*_(2, 15)_ = 4.380, *P* = 0.0318; Cg3: *F*_(2, 15)_ = 21.38, *P* < 0.0001; Figure 6]. None of the other regions analyzed by one-way ANOVA exhibited changes in CB1 receptor levels between any of the three dietary groups [caudate putamen: *F*_(2, 15)_ = 1.894, *P* = 0.1847; nucleus accumbens core: *F*_(2, 15)_ = 0.1655, *P* = *ns*; nucleus accumbens shell: *F*_(2, 15)_ = 0.0719, *P* = *ns*; CA1: *F*_(2, 15)_ = 0.3172, *P* = *ns*; CA2: *F*_(2, 15)_ = 0.3150, *P* = *ns*, CA3: *F*_(2, 15)_ = 0.7521, *P* = *ns*; dentate gyrus: *F*_(2, 15)_ = 1.167, *P* = *ns*; amygdala: *F*_(2, 15)_ = 0.3150 *P* = *ns*; ventromedial hypothalamus: *F*_(2, 15)_ = 0.9755, *P* = *ns*; lateral hypothalamus: *F*_(2, 15)_ = 0.4275, *P* = *ns*; Table 4].

**Table 4:** CB_1_ receptor density in selected areas involved in eating behavior.

	**CTRL**	**LR**	**HR**
Nucleus Accummbens (Shell)	113.9 ± 5.94	111.7 ± 5.94	111.3 ± 4.42
Nucleus Accummbens (Core)	95.88 ± 3.95	94.20 ± 5.89	91.99 ± 4.32
Caudate Putamen	142.9 ± 6.54	129.9 ± 5.40	142.5 ± 3.87
Amygdala	259.7 ± 11.57	253.4 ± 11.39	234.6 ± 29.78
Hippocampus (CA1)	514.5 ± 24.13	532.6 ± 15.99	536 ± 20.56
Hippocampus (CA2)	522.4 ± 30.61	545.3 ± 17.40	543.9 ± 17.99
Hippocampus (CA3)	478.6 ± 22.48	507.5 ± 21.23	513.1 ± 20.32
Hippocampus (gyrus dentatus)	442.4 ± 18.1	431.7 ± 25.03	477.7 ± 23.09
Hypotalamus Lateral	265.7 ± 10.04	279.2 ± 12.70	248.9 ± 36.86
Hypothalamus ventro-medial	287.8 ± 24.71	287.4 ± 15.75	240.1 ± 37.94

*Data are expressed as mean fmol/mg protein of [3H]CP55940 ± SEM (n = 6 per group). None brain regions analyzed by one-way ANOVA exhibited change in the CB_1_ receptor levels when comparing all three experimental groups*.

### Effect of binge eating behavior on plasma hormone levels

**Figure 7:**
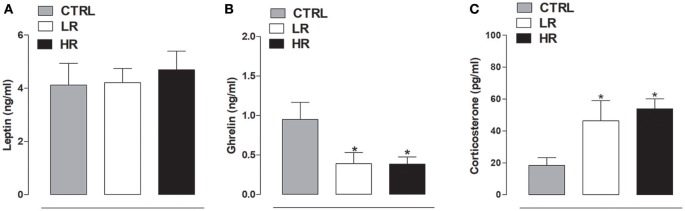
Plasma hormone levels. **(A)** Leptin: data are expressed as mean ng/ml ± SEM (*n* = 6 per group). No changes were found between all three experimental groups. **(B)** Ghrelin: data are expressed as mean ng/ml ± SEM (*n* = 6 per group). Both LR and HR groups showed a decrease of plasma ghrelin levels compared to CTRL group (one-way ANOVA: ^*^P < 0.05, Newman–Keuls multiple comparison test vs. CTRL group). **(C)** Corticosterone: data are expressed as mean pg/ml ± SEM (*n* = 6 per group). Both LR and HR groups showed an increase compared to CTRL group (one-way ANOVA: ^*^P < 0.05, Newman–Keuls multiple comparison test vs. CTRL group).

As shown in Figure 7A, no difference was found in leptin plasma levels between any of the dietary groups [one-way ANOVA: *F*_(2, 15)_ = 0.1991, *P* = *ns*]. In contrast, individual one-way ANOVA detected a significant difference in plasma ghrelin levels between the dietary groups [*F*_(2, 15)_ = 4.264, *P* < 0.05]; *post-hoc* analysis revealed both LR and HR rats had lower levels than the CTRL group (Figure 7B). Finally, corticosterone plasma levels were significantly increased in both LR and HR groups compared to CTRL rats [*F*_(2, 15)_ = 4.683, *P* < 0.05; Figure 7C].

## Discussion

The primary aim of the current study was to assess whether endocannabinoid tone could be modified in female rats exhibiting binge eating behavior, specifically in brain areas involved with food-motivated behaviors (Benoit et al., 2010; Behary and Miras, 2014; Tulloch et al., 2015; Lau et al., 2017). Binge eating behavior was induced using a limited access protocol as previously described (Scherma et al., 2013; Satta et al., 2016). Consistent with our earlier **studies**, we showed that when access to a HFD, such as margarine, become restricted to 3 days/week for 2 h (HR group), rats gradually increased their intake of margarine with initial access until finally stabilizing. On the other hand, rats on a less severe limited access schedule (7 days/week for 2 h; LR group) showed stable margarine intake over time. As reviewed by Corwin et al. (2011), in the limited access model, binge eating behavior is established when intake of the palatable food in the sporadic access group exceeds that of the daily access group. Indeed, after a few weeks, margarine intake in the HR group (binging group) became significantly greater than that in the LR group. Once the difference in margarine intake between the two groups was firmly established (by week 5), we analyzed the tissue levels of AEA, 2-AG, OEA, and PEA, as well as CB1 receptor density in various brain regions associated with food-motivated behaviors, taking samples immediately after margarine consumption on the last day of the study.

Our data clearly showed an alteration in endocannabinoid levels in the HR group: AEA significantly decreased in the caudate putamen, amygdala, and hippocampus when compared to CTRL. In contrast, 2-AG levels were significantly increased in the hippocampus. However, our study indicated that these alterations were not specific to the binging group (HR group); the LR group also showed a decrease of AEA levels in the amygdala as well as an increase of 2-AG levels in the hippocampus when compared to CTRL. Moreover, compared to HR and CTRL groups, the LR group had AEA decreased in the prefrontal cortex and increased in the nucleus accumbens. We did not observe any dietary group differences in the tissue levels of AEA and 2-AG in the hypothalamus, which plays an important role in the homeostatic regulation of food intake. It is important to emphasize that the LR group was also subjected to limited margarine access, but to a lesser extent than the HR group. Therefore, although not comparable to the HR group, margarine consumption in the LR group could account for the same results obtained in both groups. In agreement, Corwin et al. (1998) showed that non-food deprived rats with access to chow *ad libitum* will binge on a vegetable fat (shortening) when it is presented for 2 h each day, and this effect is enhanced when the fat is offered only three times per week.

The fact that alterations in the endocannabinoid levels were detected only in the animals that had access to margarine suggests that these changes are the consequence of exposure to this palatable food. Since we only run animals that were subjected to a restricted consumption design, a limitation of this study could be due to the lack of a control group with continuous access to margarine. However, previous observation showed that endocannabinoids levels change in rats fed HFD continuously relative to rats maintained on a standard diet only (Jager and Witkamp, 2014) For example, HFD treatment induced obesity in mice increased both AEA and 2-AG in the hippocampus as well as 2-AG in the hypothalamus (Massa et al., 2010; Bisogno et al., 2013). The same hypothalamic increase of 2-AG was also found in Wistar rats fed *ad libitum* with HFD for 12 weeks (Gamelin et al., 2017). Thus, our data further confirm that endocannabinoids levels are more easily influenced by exposure to HFD.

As mentioned in the introduction, all the brain areas analyzed in this study are part of the mesocorticolimbic system that represent the principal neural pathway that drive hedonic feeding (Meye and Adan, 2014; Lau et al., 2017). In agreement, it has been demonstrated that palatable foods stimulate the mesocorticolimbic dopamine system in a way similar to that of drugs of abuse, by increasing dopamine release in the shell of the nucleus accumbens (Martel and Fantino, 1996). Importantly, the increase in dopamine induced by presentation of palatable foods is blocked by administration of rimonabant (Melis et al., 2007), which suggests that the hedonic response to food might depend on the endocannabinoid system, probably through modulation of the mesocorticolimbic system. Consistent with this, acting as retrograde messengers, endocannabinoids can modulate excitatory and inhibitory inputs that control dopaminergic neurons within this system (Araque et al., 2017). Thus, it is possible that changes in endocannabinoid levels in our animals, due to margarine exposure, could determine alterations in the hedonic processes which could lead to excessive consumption of this palatable food. Further studies evaluating the dopaminergic signal in our animals are needed to validate this hypothesis. However, it has been well established that chronic exposure to a HFD induce alterations in the mesocorticolimbic dopamine system (Furlong et al., 2014; Corwin et al., 2016; Lau et al., 2017).

In both HR and LR groups, OEA was significantly decreased in the hypothalamus compared to CTRL. No difference was observed in the other brain regions analyzed. As already mentioned in the introduction, OEA has recently received much attention for its important role in the regulation of feeding behavior by acting as a mediator of satiety (Lo Verme et al., 2005; Romano et al., 2015). Indeed, OEA induces reduction of food intake and body mass by activation of peroxisome proliferator-activated receptor of type α, a major transcriptional regulator of lipid metabolism and energy balance (Fu et al., 2003). As for endocannabinoids, levels of OEA in peripheral and central tissues can be affected by several factors, including the fat content of the diet. Aviello et al. (2008) showed that mice fed a HFD for 8 weeks had lower levels of OEA in the small intestine. Moreover exposure to a HFD also reduced intestinal OEA levels in rats (Diep et al., 2011). It has been hypothesized that a decrease in OEA levels by HFDs may contribute to decreased satiety and therefore, induce hyperphagia (Romano et al., 2015). In agreement, feeding-induced OEA mobilization is disrupted in the gut of rats and mice rendered obese by exposure to a HFD (Igarashi et al., 2015). In line with these data, it is possible that the reduction of OEA levels we found in the hypothalamus could contribute to sustainment of margarine intake in both HR and LR groups. Unlike OEA, we did not find a difference in PEA levels between any of the dietary groups.

Another finding of the present study was that the consumption of margarine significantly reduced CB_1_ receptor density in the prefrontal cortex (cingulate cortex Cg1 and Cg3) in both LR and HR groups compared to CTRL rats. In agreement with our data, Bello et al. (2012) showed a significant decrease in CB_1_ receptor expression in the cingulate cortex in rats with repeated access to a highly palatable food compared to groups that had *ad libitum* feeding schedules (i.e., continuous access and naïve controls). Consistently, long-term consumption of a palatable high energy diet has been shown to significantly decrease CB_1_ receptor mRNA expression levels in the cingulate cortex (Timofeeva et al., 2009).

Herein, we did not observe any differences in the plasma levels of leptin between any of the experimental groups. By contrast, plasma analysis showed that margarine consumption was able to significantly reduce ghrelin levels in both LR and HR rats compared to CTRL. Ghrelin is a gastric-derived hormone widely known for its centrally-mediated regulation of short- and long-term energy homeostasis by increasing hunger, food intake, and adiposity (Cummings, 2006). This hunger signal also has an important role in the rewarding and motivational aspects of the consumption of palatable food by the activation of the mesolimbic dopaminergic system (Jerlhag et al., 2007; Jerlhag, 2008). In agreement, ghrelin has been found to enhance the rewarding value of a HFD when administered to *ad libitum*-fed mice (Perello et al., 2010), as well as the motivation to obtain preferred foods (King et al., 2011). In agreement, ghrelin receptor knockout mice failed to increase their high fat intake during repeated access and did not activate the mesolimbic pathway in response to HFD consumption (Valdivia et al., 2015). Considering this, plasma ghrelin levels were expected to be elevated rather than diminished in our HR and LR groups. It must be emphasized that in the present study, we took plasma samples immediately after the 2 h-access to margarine; therefore, the lowered plasma levels could be a consequence of its consumption.

Current plasma analysis also showed significantly increased levels of corticosterone in both HR and LR groups compared to CTRL rats, that could be due an increase in the activity of the hypothalamic-pituitary-adrenal axis. Consistent with this, different studies have suggested that consumption of palatable food may affect functions of the HPA axis. Indeed, in animals fed on HFD increased plasma corticosterone levels were found (Tannenbaum et al., 1997; Pratchayasakul et al., 2011) supporting the hypothesis of the role of HFD as a stressor stimulating HPA activity (Legendre and Harris, 2006). On the other hand, it has been shown that glucocorticoids induce an appetite preference for a high fat or energy diet (Liu et al., 2017).

Our present data clearly showed an alteration in endocannabinoid tone in both HR and LR groups in specific brain areas important for the perception of the hedonic properties of palatable foods, as well as for learning processes, including those related to the consequences of eating (Hsu et al., 2017; Kanoski and Grill, 2017). Even if not uniquely specific to binge eating, HFD-induced changes in the endocannabinoid tone could contributes to the to the development and maintenance of this behavior.

## Author contributions

VS performed and analyzed the behavioral and CB_1_ receptor autoradiography experiments, was involved in the discussions of the data and wrote the first draft of the manuscript. MS performed and analyzed the behavioral experiments, was involved in the discussions of the data and contributed to write the manuscript; FP performed and analyzed the lipid extraction and endocannabinoid measurements and was involved in discussions of the data. TB supervised the lipid extraction and endocannabinoid measurements, was involved in the design of the study and in discussions of the data. MC supervised the CB_1_ receptor autoradiography experiments, was involved in the design of the study and in discussions of the data; PU supervised the plasma hormone measurements, was involved in the design of the study and in the discussion of the data. WF was involved in the design of the study, discussions of the data and participated in revising the article. PF designed the research proposal, supervised the study and edited the final version of the manuscript. All authors approved the final version of the manuscript.

### Conflict of interest statement

The authors declare that the research was conducted in the absence of any commercial or financial relationships that could be construed as a potential conflict of interest.
